# Despite low viral titer in saliva samples, Sanger-based SARS-CoV-2 spike gene sequencing is highly applicable for the variant identification

**DOI:** 10.1186/s12920-023-01633-5

**Published:** 2023-08-24

**Authors:** Ko Ko, Kazuaki Takahashi, Noriaki Ito, Aya Sugiyama, Shintaro Nagashima, Kei Miwata, Yoshihiro Kitahara, Mafumi Okimoto, Serge Ouoba, Golda Ataa Akuffo, Bunthen E, Tomoyuki Akita, Toshiro Takafuta, Junko Tanaka

**Affiliations:** 1 Department of Epidemiology, Infectious Disease Control and Prevention, Graduate School of Biomedical and Health Sciences, Hiroshima University, 1-2-3, Kasumi, Minami-ku, Hiroshima, 734-8551 Japan; 2 Hiroshima City Funairi Citizens Hospital, Hiroshima, Japan; 3 Unité de Recherche Clinique de Nanoro (URCN), Institut de Recherche en Science de La Santé (IRSS), Nanoro, Burkina Faso; 4 Payment Certification Agency (PCA), Ministry of Health, Phnom Penh, Cambodia

**Keywords:** SARS-CoV-2, Sanger method, Next generation sequencing, Amplification, Variants, Screening, Japan

## Abstract

**Background:**

This study aimed to compare the performance of Sanger-based SARS-CoV-2 spike gene sequencing and Next Generation Sequencing (NGS)-based full-genome sequencing for variant identification in saliva samples with low viral titer.

**Methods:**

Using 241 stocked saliva samples collected from confirmed COVID-19 patients between November 2020 and March 2022 in Hiroshima, SARS-CoV-2 spike gene sequencing (nt22735-nt23532) was performed by nested RT-PCR and Sanger platform using in-house primers. The same samples underwent full-genome sequencing by NGS using Illumina NextSeq2000.

**Results:**

Among 241 samples, 147 were amplified by both the Sanger and the Illumina NextSeq2000 NGS, 86 by Sanger only, and 8 were not amplified at all. The overall amplification rates of Illumina NextSeq2000 NGS and Sanger were 61% and 96.7%, respectively. At low viral titer (< 10^3^ copies/mL), Illumina NextSeq2000 NGS provided 19.2% amplification, while Sanger was 89.7% (*p* < 0.0001). Both platforms identified 38 wild type, 54 Alpha variants, 84 Delta variants, and 57 Omicron variants.

**Conclusions:**

Our study provided evidence to expand the capacity of Sanger-based SARS-CoV-2 spike gene sequencing for variants identification over full-genome by Illumina NextSeq2000 NGS for mass screening. Therefore, the feasible and simple Sanger-based SARS-CoV-2 spike gene sequencing is practical for the initial variants screening, which might reduce the gap between the rapid evolution of SARS-CoV-2 and its molecular surveillance.

## Introduction

The novel Coronavirus disease (COVID-19) pandemic, which started in December 2019 [1], is a global concern due to its rapid evolution and the emergence of new variants that change its virulence, transmission power, infectivity, and so forth. Despite the development of a variety of vaccines against SARS-CoV-2, many reported breakthrough infections threaten not only the vaccine escapes but also the diagnostic escapes of the virus.

The SARS-CoV-2 virus is a positive single-stranded RNA virus with 30,000 base pairs and has several Open Reading Frames (ORFs). The Spike protein (S) of SARS-CoV-2 aids in viral entry into its host cells. The S protein is composed of 1,273 amino acids. It consists of two subunits: S1, which is vital for the virus binding to the host cell receptor ACE-2 [2], and S2, which aids in merging the virus with the cell membranes of the host [3]. Until now, the sequential emergence of SARS-CoV-2 variants of concern defined by the World Health Organization (WHO) occurred, including Alpha (B.1.1.7), Beta (B.1.351), Gamma (P.1), Delta (B.1.617.2), Omicron (B.1.1.529), and the sub-lineage of Omicron variant (BA.1, BA.2, BA.3, BA.4, BA.5) [4] and recombinant (XD, XE and XF), all of which have different dynamics and multiple polymorphisms on the Spike gene [5].

To control the pandemic, it is essential to implement effective sampling and diagnosis methods. The nasopharyngeal (NPS) or oropharyngeal swabs (OPS) are the gold standard sampling strategy for droplet or airborne infections. However, they have disadvantages such as high invasiveness, need for skilled health care personnel (HCPs), bleeding, patient discomfort, and high infection risk to HCPs [6]. The saliva sampling strategy becomes the alternative or substitute to NPS or OPS because of the reported high sensitivity (80.3%) and specificity (99.4%) for detection of SARS-CoV-2 [7], less invasiveness, self-collection, removal of infection risk to HCPs, and convenient and easy sampling strategy for children.

At the pandemic’s start, simple and rapid methods were used for mass screening and diagnosis [8, 9]. This includes nonspecific laboratory detection methods like D-dimer, Ferritin, LDH estimation, molecular testing, antigen, and serological tests [1]. Antigen-based immunoassays and ELISA are reliable methods for detecting the virus [10]. Antibody-based tests also use binding assay procedures like immunofluorescence and immunochromatographic assays to detect viral antibodies. Nevertheless, real-time reverse-transcriptase polymerase chain reaction (RT-qPCR), which detects the specific viral RNA of the spike, envelope, nucleocapsids or ORF1-ab regions, is denoted as the standard test for detection of SARS-CoV-2 virus [1, 8]. However, these tests mentioned earlier cannot be utilized for variant identification.

RT-qPCR is used to detect the viral RNA regions, but this method has some inaccuracies due to viral mutations [8]. Loop-Mediated Isothermal Amplification (LAMP), a technique that gained a lot of attention during the SARS-CoV-2 pandemic, uses about six primers to form loops of continuous isothermal deoxy-/ribonucleic acid (DNA/RNA) replication. It is a short method that can be performed at the point of care [11] and has been used alternatively for variant identification. The Clustered Regularly Interspaced Short Palindromic Repeats (CRISPR) have also been utilized in variant identification [8]. This method relies on type V CRISPR-Cas12, or type VI CRISPR-Cas13, both enzymes, exhibit nonspecific endonuclease activity in trans after binding to a specific cis target via programmable CRISPR RNAs (crRNAs) by combining isothermal amplification methods [12]. Genome tiling array is a cheap, reliable, and accurate rapid method for surveillance of rapidly mutating SARS-CoV-2 viral strains [13]. A highly accurate chip-based resequencing method for SARS-CoV-2 variant identification had been developed, which was adopted from DNA arrays, a method used in detecting, surveillance, and screening multivariant strains [9].

Despite the numerous options developed for variant identification, the Next Generation Sequencing (NGS) remains the gold standard for the identification of new SARS-CoV-2 variants [14]. Various technologies and platforms for NGS, such as Illumina NextSeq2000, iSeq 100, MiniSeq, etc., have been developed and heavily relied on for variant surveillance worldwide. However, the amplification power, requirement of advanced technology, high-quality resources, and high cost are challenges in their utilization [15].

Reliance on the NGS alone has resulted in a deficit in the number of tests in the Global Initiative on Sharing Avian Influenza Data (GISAID) database of about 13 million registered strains compared to about 600 million cases recorded. This can be rectified by adopting the Sanger method, which has been used to identify essential SARS-CoV-2 variants without the need for whole genome sequencing [14, 16–18]. The Sanger method is a technique of complete nucleotide sequencing of the viral RNA, which can give enough data about the morphisms of the virus without the need for whole genome sequencing [3, 14, 16–18]. Hence, it can quickly be adopted in any laboratory with experience in sequencing. The Sanger technology is more affordable and accessible and can accurately screen significant variants associated with the new outbreak. Furthermore, it has a short sample processing time. The nucleotide sequence of of the Receptor Binding Domain (RBD) region in a typical research set-up using the Sanger method could be analyzed in a maximum of 2 days [17, 19]. Hence, this study aimed to compare the performance of Sanger-based SARS-CoV-2 spike gene sequencing and NGS-based full-genome sequencing for variant identification in saliva samples with low viral titer.

## Materials and method

This study included 241 stocked saliva samples collected from all the confirmed COVID-19 patients admitted to F Hospital in Hiroshima, Japan, between November 18, 2020, and March 15, 2022. F hospital is one of the main COVID-19 treatment centers in the whole Hiroshima prefecture. The samples were collected for the molecular surveillance of the SARS-CoV-2 virus to perform the mutation screening and the variant identification of SARS-CoV-2 in Hiroshima.

The saliva samples were self-collected in a 5 mL sterile container using the passive drool method under the instruction and observation of healthcare personnel, and the samples were temporarily stored at 4℃ for less than 12 h and transported in the cold chain from the sampling site to the laboratory at the end of the day. Then the samples were kept at -80℃ until analysis.

The SARS-CoV-2 ribonucleic acid (RNA) was extracted by the SMI-TEST R&D kit (Medical and Biological Laboratories Co. Ltd., MA, USA) as described in detail in our previous reports [16, 17, 20]. The final pellet of the extracted RNA was dissolved in 50μL of RNase free water and kept at -80℃ until analysis. Then the viral titer was determined using 10% (5μL) of the extracted template RNA mixed with nucleocapsid (N) protein-specific primers NIID_2019-nCoV_N_F2 [5’AAATTTTGGGGACCAGGAAC3’] and NIID_2019-nCoV_N_R2 [5’TGGCAGCTGTGTAGGTCAAC3’], and probe NIID_2019-nCoV_N_P2 [5’FAM-ATGTCGCGCATTGGCATGGA-BHQ3’] [21] in Step One Real-Time PCR System (Thermo Fisher Scientific, Applied Biosystems, Foster City, CA, USA) as previously reported [16, 17, 20]. The measured values of viral titer were transformed into the number of copies per milliliter.

The partial spike genome of SARS-CoV-2 virus targeting nucleotide position 22735 to 23532, was amplified by two rounds of the nested Reverse Transcriptase-Polymerase chain reaction (nested RT-PCR) [22] with the in-house developed primer sets (22632S: 5'GAATCAGCAACTGTGTTGCTG3', 22659S: 5'CTGTCCTATATAATTCCGCATC3', 22659_Omi: 5'CTGTCCTATATAATCTCGCACC3', SP35AS: 5'TGACTAGCTACACTACGTGC3' and SP36AS: 5'TTAGTCTGAGTCTGATAACTAG3' for 1^st^ round of nested RT-PCR, 22687S: 5'CACTTTTAAGTGTTATGGAGTG3', 22712S: 5'CCTACTAAATTAAATGATCTCTG3', SP37AS: 5'GCATATACCTGCACCAATGG3' and SP38AS: 5'TATGTCACACTCATATGAGTTG3' for 2^nd^ round of nested PCR). Then the amplicon was sequenced by SeqStudio Sequence Analyzer (Thermo Fisher Scientific, Applied Biosystems, Foster City, CA, USA) with the primers 22712S and SP38AS. The detailed procedures for the amplification and sequencing by the Sanger method were described in our previously published report [16].

All the same samples underwent full genome sequencing by NGS strategy using the Illumina NextSeq2000 (Illumina Inc., California, USA) at the Repository of Data and Biospecimen of Infectious Disease (REBIND: https://rebind.ncgm.go.jp/About) founded by the Pathogen Genomics Center, National Institute of Infectious Diseases, Tokyo, Japan. After the RNA extraction by QIAamp Viral RNA Mini Kit (Qiagen, USA), cDNA was synthesized. Library construction was done by the Illumina COVIDSeq™ test (Illumina, California, USA) and then sequenced using sequencing by synthesis (SBS) on NextSeq 2000 system.

The homology between the partial spike gene obtained by the Sanger method and the corresponding spike region of the full SARS-CoV-2 genome in the same samples was examined by assembling the sequences in Genetynx Mac v21 (Genetynx Corporation, Tokyo, Japan). The proportion of sequencing by each platform was calculated using Microsoft Excel (Microsoft Corporation, New Mexico, USA). The comparison of the amplification between Sanger and NGS platforms was examined by nonparametric comparison for all pairs using the Steel–Dwass method, and the 2-way ANOVA was applied to compare the amplification rate of NGS and Sanger platforms in different SARS-CoV-2 variants using JMP ver.16 (SAS Institute Inc., Cary, NC, USA).

A total of 5,533 SARS-CoV-2 full-genome sequences of Hiroshima were retrieved from the Global Initiative on Sharing All Influenza Data (GISAID: http://www.gisaid.org), including 147 SARS-CoV-2 full genome sequences of this study registered through REBIND. All the sequences data were submitted from Hiroshima Prefecture, Japan, between March 13, 2020, and September 25, 2022. Then, these 5,533 SARS-CoV-2 full-genome sequences, representing the whole of Hiroshima Prefecture, were subjected to evolutionary analysis by the Unweighted Pair Group Method with Arithmetic mean (UPGMA) method using Molecular Evolutionary Genetics Analysis (MEGA) version X [23].

## Results

**Fig. 1:**
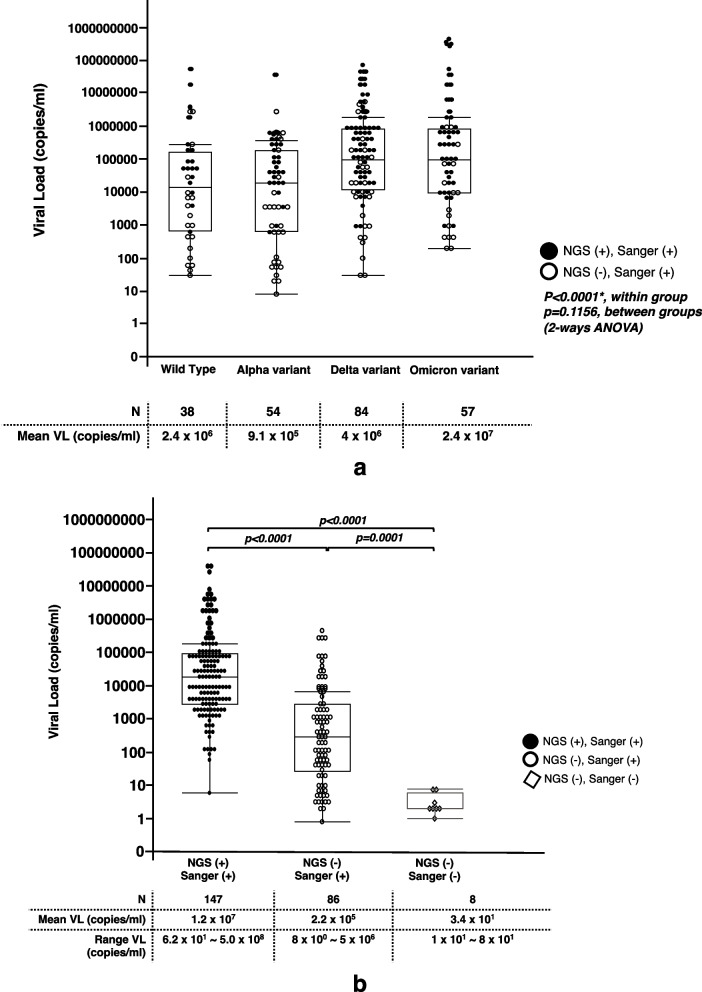
**a** Distribution of SARS-CoV-2 viral titer in 241 saliva samples stratified by SARS-CoV-2 variants. This figure shows that the SARS-CoV-2 viral titers differed by the variant types, where the black circle spots represent Illumina NextSeq2000 NGS ( +) and Sanger ( +), the white circle spots represent Illumina NextSeq2000 NGS (-) but Sanger ( +). **b** Distribution of SARS-CoV-2 viral titer in 241 saliva samples stratified by amplification status. This figure shows that SARS-CoV-2 viral titer differed by the amplification status of the Sanger platform and Illumina NextSeq2000 NGS platform. The black circle spots represent the samples amplified by both the Sanger and the Illumina NextSeq2000 NGS platforms, the white circle spots for the samples amplified only by the Sanger platform, and the white diamond spots represent the samples that were not amplified at all by both platforms

A total of 241 stocked saliva samples from all the confirmed COVID-19 patients at the study hospital in Hiroshima during the study period were included. The distribution of SARS-CoV-2 viral titer among all 241 samples by SARS-CoV-2 variants is shown in Fig. 1a. During the SARS-CoV-2 wild-type domination, the viral titer in 38 SARS-CoV-2 wild-type samples ranged from 3 × 10^1^ to 6 × 10^8^ copies/mL, with a mean viral titer of 2.4 × 10^6^ copies/mL. During the SARS-CoV-2 Alpha variant domination, the viral titer in 54 Alpha variant samples ranged from 8 to 4 × 10^7^ copies/mL with a mean titer of 9.1 × 10^5^ copies/mL. Then, during the SARS-CoV-2 Delta variant domination, the viral titer among 84 Delta variant samples ranged from 3 × 10^1^ to 8 × 10^7^ copies/mL, with a mean titer of 4 × 10^6^ copies/mL. Finally, during SARS-CoV-2 Omicron variant domination, the viral titer among 57 Omicron variant samples ranged from 2 × 10^2^ to 5 × 10^8^ copies/mL, with a mean titer of 2.4 × 10^7^ copies/mL.

By 2-way ANOVA test, there was a significant difference in the amplification rates by Illumina NextSeq2000 NGS and Sanger in all variants within the groups (*p* < 0.0001), but no significant difference was found between variants (*p* = 0.1165) (Fig. 1a).

Grouping the samples by positive amplification by the two sequencing platforms, 147 samples were amplified by both the Sanger and Illumina NextSeq2000 NGS method, 86 samples were amplified only by the Sanger method, and 8 samples were not amplified at all by both sequencing platforms. The viral titer ranged from 60 to 5 × 10^8^ copies/mL, with a mean titer of 1.2 × 10^7^ copies/mL among the 147 samples successfully amplified by both platforms. It ranged from 8 to 5 × 10^6^ copies/mL, with a mean titer of 2.2 × 10^5^ copies/mL among the 86 samples amplified only by the Sanger method. For the 8 samples with negative amplification by both platforms, the viral titer was between 10 and 80 copies/mL, with a mean titer of 34 copies/mL. A significant difference in viral titer in each group was found (*p ≤* 0.0001, Steel–Dwass test) (Fig. 1b).

To further assess the amplification rates of the two platforms, the samples were divided into three groups based on their viral titer: low viral titer (≤ 10^3^ copies/mL), intermediate viral titer (between 10^4^ and 10^6^ copies/mL), and high viral titer (≥ 10^7^ copies/mL). Among 20 samples with a high viral titer, the amplification rate of Illumina NextSeq2000 NGS was 90%, whereas that of the Sanger platform was 100%. In the 143 samples with an intermediate viral titer, the amplification rate of Illumina NextSeq2000 NGS was 79.7%, and that of the Sanger platform was 100%. Regarding the 78 samples with a low viral titer, the amplification rate of Illumina NextSeq2000 NGS was 19.2%, while that of the Sanger platform was 89.7%. The amplification rates between the Illumina NextSeq2000 NGS and the Sanger platform significantly differed in each group (*p* < 0.0001) (Table 1).

**Table 1:** Frequency of sequences obtained by Illumina NextSeq2000 NGS and Sanger stratified by viral titer

**Viral titer (copies/mL)**	**N**	**Amplification by Illumina NextSeq2000 NGS**			**Amplification by Sanger platform**			***p*-value**
		**Detected**	**Undetected**	**% Detected**	**Detected**	**Undetected**	**% Detected**	
≤ 10^3^	78	15	63	**19.2%**	70	8	**89.7%**	** < 0.0001**
10^4^ ~ 10^6^	143	114	29	**79.7%**	143	0	**100%**	** < 0.0001**
≥ 10^7^	20	18	2	**90%**	20	0	**100%**	0.0793
Total	241	147	94	**61%**	233	8	**96.7%**	** < 0.0001**

In our study, among 244 samples, 233 samples were positively amplified and identified SARS-CoV-2 variants, among which 147 were identified by both the Illumina NextSeq2000 NGS and the Sanger methods. Therefore, 38 samples were identified as wild-type, 54 samples were Alpha variant, 84 were Delta variant, and 57 were Omicron variant (BA.1). The percentage of identification by each method is shown in Table 2, and the identification rate was significantly higher in the Sanger method.

**Table 2:** Identification of variants by Illumina NextSeq2000 NGS and Sanger method

**Variant Type**	**N**	**Identified by Illumina NextSeq2000 NGS**		**Identified by Sanger**		***p*-value**
		**n**	**%**	**n**	**%**	
Wild Type	38	20	**52.7%**	38	**100%**	** < 0.0001**
Alpha Variant	54	28	**51.9%**	54	**100%**	** < 0.0001**
Delta Variant	84	57	**67.8%**	84	**100%**	** < 0.0001**
Omicron Variant (BA.1)	57	42	**73.7**	57	**100%**	** < 0.0001**
Total	233	147	**61%**	233	**100%**	** < 0.0001**

**Fig. 2:**
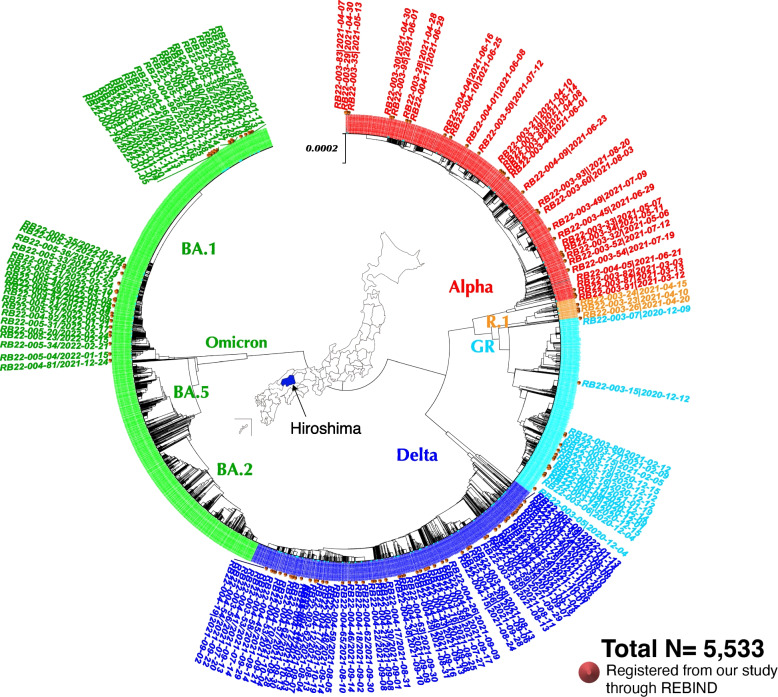
Phylogenetic tree of SARS-CoV-2 strains retrieved from GISAID, including 147 strains registered by our study through REBIND. This phylogenetic tree contains 5533 SARS-CoV-2 strains retrieved from GISAID, all of which were submitted from Hiroshima, Japan, including 147 samples from this study that are shown with brown spots stating the sample ID and collection date. The light blue color represents the SARS-CoV-2 wild type, the red for the Alpha variant, the dark blue for the Delta variant, and the green for the Omicron variant

The phylogenetic tree showed that the wild-type (Nextstrain Clade 20B) was dominant until early January 2021, then the lineage R.1 was found in March and April 2021. Immediately after, the Alpha variant (Nextstrain Clade 20I) became dominant until mid-August 2022, followed by the Delta variant (Nextstrain Clade 21 J) until November 2022. In December 2022, Delta and Omicron variants (Nextstrain Clade 21 K) were found. After January 2022, the Omicron variant was solely dominant, causing a massive outbreak in Hiroshima, and all those Omicron variants belonged to the sub-lineage BA.1. The tree also revealed the existence of subvariants or sub-lineages under the main variants of concern. The outbreak pattern of the Alpha, Delta, and Omicron variants (BA.1) was quite different. The Delta variant caused many small clusters of cases with slightly different sub-lineages than the Alpha variant. The Omicron sub-lineage BA.1 caused a big cluster of cases from the same source, while the Omicron sub-lineage BA.2 and BA.5 caused many small clusters of cases like the Delta variant (Fig. 2).

## Discussion

This study compared the amplification rates of two different sequencing platforms, namely partial genome sequencing by the Sanger method and full genome by Illumina NextSeq2000 NGS, from the perspective of massive screening and SARS-CoV-2 variants identification. There are some reports on the comparison between the NGS and the Sanger strategies for mutation screening and genotype identification of hepatitis B virus (HBV) [24] and hepatitis C virus (HCV) [25], but reports on the SARS-CoV-2 virus are limited. Hence, a variety of methods were developed to identify the variants of concern, such as amplicon-based whole or partial genome sequencing, multiplex real-time polymerase chain reaction (RT-PCR) assays [26], S-gene target failure (SGTF) [27], screening single nucleotide polymorphism (SNP) assays [28], reverse transcription loop-mediated isothermal amplification (RT-LAMP) [29], transcription-mediated amplification (TMA) [30], among others. Quantitative real-time reverse transcription polymerase chain reaction (RT-qPCR) is the gold standard for detecting the SARS-CoV-2 virus and is a useful tool for diagnosis or confirmation because of its high accuracy and sensitivity, but it cannot identify the variants of concern. The S-gene target failure (SGTF) using the ThermoFisher TaqPath™ COVID-19 Combo kit was developed and has a sensitivity of 92% and a specificity of 98% to detect the Omicron variant (B.1.1.529) sub-lineage BA.1/BA1.1 [31]. However, the rapid emergence of new virulent strains under the same lineage challenges the generalization of its usefulness because it is designed to detect the particular variant of concern and cannot identify other variants. The gap between the rapid emergence of new SARS-CoV-2 variants and the feasible screening system for SARS-CoV-2 variants identification is still unsolved.

In our study, we assessed the performance and validation of partial genome sequencing by the Sanger platform against full genome sequencing by Illumina NextSeq2000 NGS for the SARS-CoV-2 variants identification. Though the Illumina NextSeq2000 NGS full genome sequencing provides a complete configuration on the genomic characteristics of the SARS-CoV-2 virus, besides its high cost, the main problem is that samples with Ct > 30 (low viral titer) are difficult to amplify [32, 33]. Moreover, it requires advanced technology, financial support, and human resources, which limits its access in developing countries [33, 34]. Additionally, the genome data by the Illumina NextSeq2000 NGS contains many ambiguous nucleotides (N) and somehow approximately 16% loss of nucleotide data from the original template [33].

Our study provided evidence of constructing partial genomes using the Sanger platform to identify the SARS-CoV-2 variants of concern. The strategy is based on the distinct genomic characteristics of each variant of concern in the targeted spike region (nt22735 to nt23532: 798 nucleotides) covering a part of the RBD in the S1 sub-unit [16, 17]. This area is a unique checkpoint for all variants of concern, including Alpha (B.1.1.7), Beta (B.1.351), Gamma (P.1), Delta (1.617.2), and Omicron (B.11.529) [16, 17]. In addition, the strategy allows for identifying the sub-lineages of the Omicron variant (BA.1, BA.2, and BA.3).

In our study, all 241 samples underwent SARS-CoV-2 variants identification by both the Illumina NextSeq2000 NGS and the Sanger platforms. The amplification rate by the Illumina NextSeq2000 NGS was lower than the Sanger platform if the samples had a viral titer below 10^3^ copies/mL (19.2% vs. 89.7%, *p* < 0.0001) (Table 1). This result indicates the limitation of using NGS for mass screening of SARS-CoV-2 variants and suggests that the Sanger platform is more advantageous than the Illumina NextSeq2000 NGS for mass screening of SARS-CoV-2 variants.

Our study validated and reported a significant difference in the amplification rates between the Illumina NextSeq2000 NGS and the Sanger platforms for identifying SARS-CoV-2 variants of concern. Although the NGS is the gold standard for variant identification and in-depth understanding of molecular characteristics, evolutionary changes, and mutation patterns of SARS-CoV-2, it is not practically applicable for the screening or the identification of SARS-CoV-2 variants of concern during massive outbreaks. As of December 2022, there were 646 million confirmed COVID-19 cases worldwide, but the SARS-CoV-2 full genomes submitted to GISAID were 14 million strains, representing only 2.2% of all cases worldwide. Considering other challenges and limited access to NGS in developing and resource-limited countries, the submission rates of SARS-CoV-2 full genomes in developed countries such as the UK, USA, Japan, and France were 12.1, 4.5%, 1.9%, and 1.4% respectively. The technical and resources limitation of NGS cannot be excluded.

Although NGS is a high-throughput method and the processing time from extraction to sequences using NGS may vary but it typically takes several days to 10 days. In comparison, the Sanger platform takes two to three days to complete the analysis. Therefore, the processing time is comparably short in Sanger platform, but it may depend on the facilities’ workload, sample size, and the NGS methods used. In term of the cost per sample, the NGS (approximately 25,000¥ or 175USD per sample) is considerably more expensive than Sanger platform (approximately 2,300¥ or ~ 16USD per sample), but the cost may be comparably reduced in using NGS in case of large-scale sequencing project. However, considering the processing time and cost per sample, the Sanger platform is superior to NGS, and it is very effective and functional for molecular surveillance of SARS-CoV-2 infection.

Our study demonstrated a better amplification rate by the Sanger platform than the NGS, especially among samples with low viral titers. But the comparison was between the Sanger platform and the Illumina NextSeq2000 NGS only. There are a variety of NGS technologies and systems, such as Illumina MiniSeq, NovaSeq6000, HiSeqX, MiSeq, iSeq100, etc., and further studies are needed to examine and compare the performance of other NGS platforms with the Sanger technology for SARS-CoV-2 variant screening.

Monitoring and surveillance of virus evolution are important, especially for the SARS-CoV-2 virus, whose rapid evolution rate and emergence of new variants impose the need for molecular surveillance. Our partial genome strategy by the Sanger platform is supposed to be a tool for initial screening and continuous monitoring of SARS-CoV-2 variants of concern over time. The high amplification rate supported the capacity of our strategy for mass screening of the samples. Our strategy allows the direct visualization of the genomic sequences of the targeted spike region, a single test for each sample is quite enough to identify SARS-CoV-2 variants. Moreover, our study showed no mismatch in the genomic amplification between the Sanger and the Illumina NextSeq2000 NGS platforms. All variants identified by the Illumina NextSeq2000 NGS were 100% the same as our strategy, although we could not identify the sub-lineage of the SARS-CoV-2 Delta variant.

## Conclusions

In conclusion, our study provided evidence to expand the capacity of Sanger-based SARS-CoV-2 spike gene sequencing for variants identification over full-genome by Illumina NextSeq2000 NGS for mass screening. Therefore, the feasible and simple Sanger-based SARS-CoV-2 spike gene sequencing is practical for the initial variants screening, which might reduce the gap between the rapid evolution of SARS-CoV-2 and its molecular surveillance.

## Data Availability

All full genomes sequence data of SARS-CoV-2 included in this study are deposited at GISAID (http://www.gisaid.org) and it is openly accessed with the accession numbers: *EPI_ISL_11823542* to *EPI_ISL_11823688)* or are available from the corresponding author upon the reasonable request.

## Abbreviations

COVID-19: Coronavirus Disease 2019
SARS-CoV-2: Severe Acute Respiratory Syndrome Coronavirus 2
WHO: World Health Organization
NPS: Nasopharyngeal Swab
OPS: Oropharyngeal Swab
RT-qPCR: Real Time Reverse Transcriptase Polymerase Chain Reaction
Nested RT-PCR: Nested Reverse Transcriptase Polymerase Chain Reaction
NGS: Next Generation Sequencing
SNP: Single Nucleotide Polymorphism assays
SGTF: S-gene Target Failure
LAMP: Loop-Mediated Isothermal Amplification
TMA: Transcription mediated amplification
RBD: Receptor Binding Domain
RNA: Ribonucleic Acid
